# DPPH Measurement for Phenols and Prediction of Antioxidant Activity of Phenolic Compounds in Food

**DOI:** 10.3390/cimb48010012

**Published:** 2025-12-23

**Authors:** Riku Kato, Chihiro Tada, Moeka Yamauchi, Yuto Matsumoto, Hiroaki Gotoh

**Affiliations:** Department of Chemistry and Life Science, Yokohama National University, Hodogaya-ku, Yokohama 240-8501, Japan

**Keywords:** DPPH, antioxidant capacity, QSAR, machine learning, food informatics, phenol

## Abstract

Consuming foods with high antioxidant capacity is considered beneficial to health, and predicting the antioxidant capacity of food components is important. In the 2,2-diphenyl-1-picrylhydrazyl (DPPH) assay, multiple reactions occur simultaneously, and because the experimental conditions are not standardized across studies, quantitative prediction of DPPH activity is difficult. In this study, we qualitatively and quantitatively predicted the DPPH activity of phenols in food using data obtained under unified experimental conditions and machine learning. We measured DPPH activity of 96 compounds to create a dataset comprising measurements of 274 compounds, including values previously reported by our laboratory. The classification model implemented using LightGBM showed high performance, achieving an accuracy of 0.88 and an F1 score of 0.86. The support vector regression model satisfied the Golbraikh–Tropsha criteria, with an R^2^_test_ of 0.70, RMSE_test_ of 0.44, q^2^ of 0.61, and RMSE_validation_ of 0.46. Furthermore, the chemical validity of the prediction was confirmed by comparing the results of the machine learning model with those of previous studies. This method provides a basis for the quantitative prediction of DPPH activity of numerous phenolic compounds in foods and is expected to contribute to the elucidation of the antioxidant capacity of foods.

## 1. Introduction

Reactive oxygen species (ROS) are highly reactive and promote lipid oxidation in foods. Furthermore, within the body, they are generated from oxygen taken up during respiration. When produced in excess, they attack biomolecules and cause various diseases [1,2,3]. Antioxidants suppress the generation and reactivity of ROS, thus preventing oxidation [4]. They are used as additives to prevent oxidation and quality deterioration of food. Antioxidants can be easily obtained from everyday meals. People who consume foods rich in antioxidants have a lower risk of mortality from cardiovascular, cardiac, and cerebrovascular diseases than those who do not [5,6]. Obtaining antioxidants through food is an important factor for extending healthy life expectancy. When antioxidants are obtained from food, it is necessary for the compounds to be absorbed into the body. Lipinski et al.’s Rule of Five [7] is often used as an indicator of whether a compound is easily absorbed by the body. Polyphenols are the primary antioxidants present in food. There is a correlation between the polyphenol content of commonly consumed foods and their antioxidant capacities [8]. Therefore, to elucidate the antioxidant capacity of food, it is important to understand the antioxidant properties of phenolic compounds that are easily absorbed by the body.

The 2,2-diphenyl-1-picrylhydrazyl (DPPH) assay is used to measure the ability of a substance to scavenge DPPH radicals [9]. Although various methods have been proposed to evaluate antioxidant capacity [10], no single universal method exists. The DPPH assay is widely used because it is inexpensive and provides rapid results. Although in vitro antioxidant activity measurement approaches are considered to have conceptual and technical limitations, they are considered effective for confirming whether a compound possesses antioxidant activity or not [11]. When comparing the DPPH activity of food extracts with the cumulative DPPH activity of the antioxidants contained in the food, the sum of the activities of the individual components do not match the activity of the extract [12,13,14]. However, certain studies have shown a strong correlation between the content of antioxidants (particularly phenols) and the DPPH activity of food extracts [15,16,17,18], indicating that the cumulative DPPH activity of food components can be used, to some extent, to predict the overall DPPH activity of a food. However, foods contain numerous components that are difficult to isolate for DPPH activity measurement. Therefore, predicting the DPPH activity of food components is important for understanding the DPPH activity of foods.

Regarding the structure–activity relationship (SAR) of antioxidants, several studies have reported correlations with thermodynamic indicators, such as the ionization potential (IP) [19]. Most SAR studies have compared compounds with common structures based on differences in their partial structures [20,21,22]. However, these studies have often reported inconsistent trends. One reason for this is the lack of standardized measurement conditions. When comparing DPPH assay results obtained under different experimental conditions, such as temperature and solvent, simple structural rules do not apply. As the measurement temperature increases, the observed DPPH activity increases owing to the higher kinetic energy of the reacting molecules and the greater degree of hydroxyl-group dissociation. In addition, the reaction mechanism depends strongly on solvent polarity in nonpolar solvents, reactions via the single electron transfer (SET) or sequential proton-loss electron transfer (SPLET) mechanisms are unlikely, and the hydrogen atom transfer (HAT) mechanism tends to dominate; whereas, in polar solvents, the SET or SPLET mechanism becomes more favorable, leading to changes in the measured values. The influence of these experimental variations on the measured values is particularly pronounced for molecules with a weak DPPH activity [23]. As the experimental conditions were not standardized, it was difficult to compare the results between different laboratories [24]. In this milieu, efforts have been made to standardize experimental conditions and minimize errors in the measurement results among different laboratories [25,26]. Building on this effort, a reagent company has produced a simple kit that has been in the market since 2019. Several studies have performed component analyses using this kit [27,28,29,30]. It should be noted that the reported trends only apply to a limited range of compounds. Simple structural rules may not be applicable to complex molecules, resulting in different trends observed across multiple studies. For example, it is generally known that molecules with a greater number of hydroxyl groups exhibit a higher antioxidant activity [31]. However, carnosic acid, which has two phenolic hydroxyl groups, has been reported to show a higher antioxidant activity than rosmarinic acid, which contains four phenolic hydroxyl groups [32]. Furthermore, when phenols form dimers through o–o homocoupling, the number of phenolic hydroxyl groups increases, which generally enhances DPPH activity. Nevertheless, some dimers exhibit lower DPPH activity than their monomers [33]. We believe that applying machine learning to capture more complex patterns would be beneficial.

Properties of compounds have been predicted using SAR models, such as the Hansch and Hammett equations. In recent years, machine learning, represented by neural networks (NNs), has been used to predict nonlinear relationships [34,35,36,37,38]. The primary methods for improving prediction accuracy include selecting an appropriate machine learning model and choosing suitable explanatory variables. NNs are among the best suited models for capturing nonlinear relationships. In predicting DPPH activity, some studies have collated experimental data from the literature and performed predictions using a multi-perceptron NN [39]. However, owing to the lack of standardization in measuring DPPH activity, the data are not suitable for use as training data or target values in prediction. Furthermore, reports indicate that for data that are difficult to obtain in large quantities, non-deep learning models such as gradient decision trees yield higher prediction accuracy than deep learning models such as NNs [40]. Additionally, NNs have a high degree of black-box nature, which makes it difficult provide a chemical interpretation of the predictions [41]. Regarding the explanatory input variables, reports also show that incorporating quantum chemical calculation values, not just molecular structural information, improves prediction accuracy [42]. Therefore, to ensure prediction accuracy while enabling chemical interpretation, it is advisable to use molecular descriptors, including quantum chemical calculation values, as explanatory variables, and employ machine learning models capable of importance analysis for prediction.

**Figure 1:**
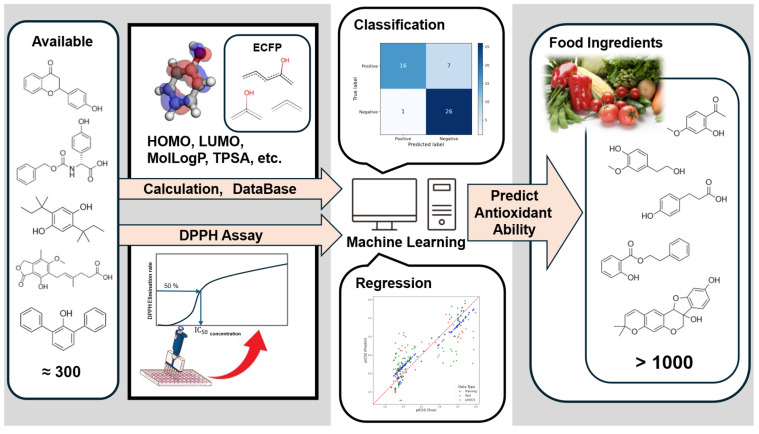
Workflow of the study.

The purpose of this study was to establish a foundation for understanding the antioxidant capacity of phenols in foods by analyzing numerous phenolic compounds using machine learning. An overview of the study workflow is presented in Figure 1. First, as predictive experimental data, the DPPH activities of various phenols were measured under standardized experimental conditions to enable comparison with previously reported DPPH activities by our group. Quantum chemical calculation values were obtained for the measured compounds and the compounds registered in FooDB [43], a dataset of food-component compounds. Furthermore, using the obtained molecular structural information and quantum chemical descriptors as explanatory variables, machine learning models were used to predict the presence or absence of DPPH activity of FooDB compounds using LightGBM [44], and to predict IC_50_ values for selected compounds using support vector machine (SVM). By analyzing the machine learning models, we determined the factors influencing DPPH activity based on the calculated molecular descriptors, including compound substructures and quantum chemical calculation values input into the models during prediction. This research is expected to deepen our understanding of the antioxidant capacity of food components that are difficult to isolate by enabling antioxidant capacity prediction without conducting experiments.

## 2. Materials and Methods

### 2.1. Reagents and Synthesis

**Figure 2:**
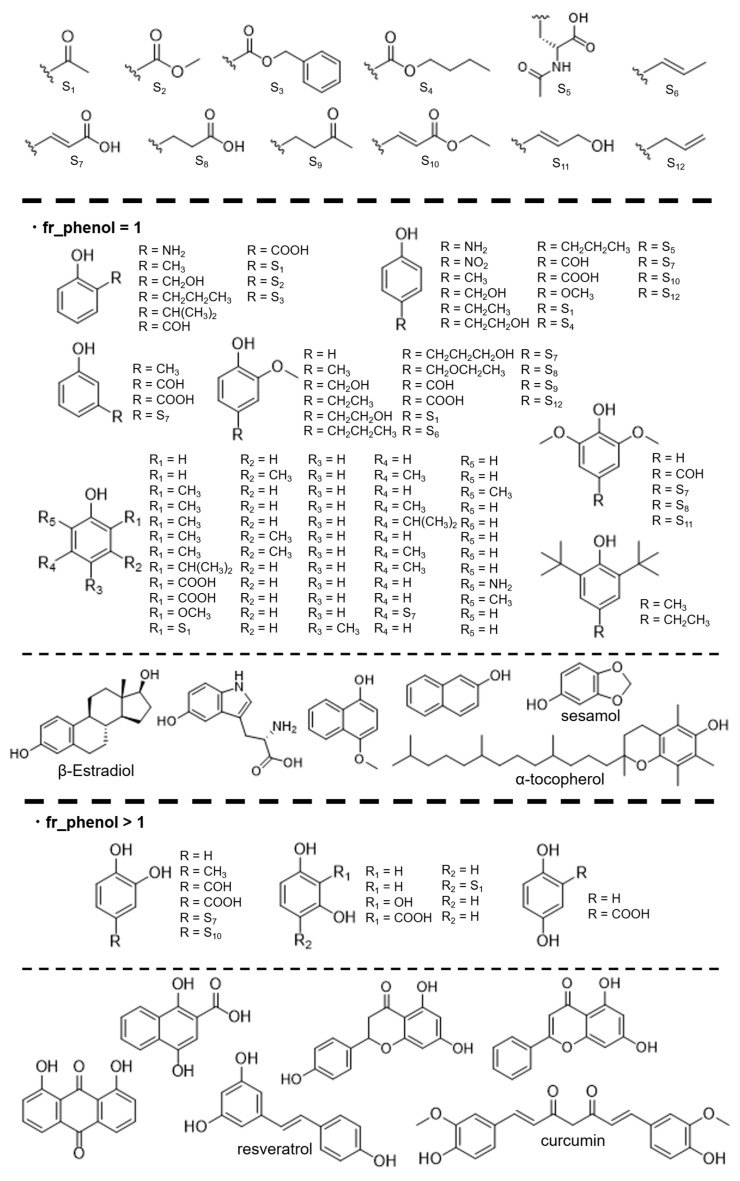
FooDB [43]-registered compounds analyzed in this study.

The reagents used in this study were purchased from Wako Pure Chemical Co. (Osaka, Japan), Tokyo Chemical Industry Co. (Tokyo, Japan), Sigma-Aldrich (St. Louis, MO, USA), Kanto Chemical Co., Inc. (Tokyo, Japan), and Thermo Fisher Scientific (Waltham, MA, USA). For compounds with only one hydroxyl group, reagents were selected to cover a range of compounds with structures similar to those registered in FooDB. The reagents were then analyzed without purification. Furthermore, as eight compounds could not be purchased directly, they were synthesized according to previously reported methods [45]. These compounds were selected to fill the gaps in the physical properties and structural space in our previously reported measurement data [19,33] and food component compounds, resulting in a total of 96 measured compounds. Some of these data are shown in Figure 2. The details of these compounds are provided in the Supplementary Information (SI.xlsx).

### 2.2. DPPH Radical-Scavenging Activity Measurement

The DPPH radical-scavenging activity was measured using the DPPH Antioxidant Assay Kit from Dojin Chemical Laboratory (Kumamoto, Japan) [46] following the manufacturer’s protocol. The total reaction volume was 200 µL, consisting of 100 µL (50% (*v*/*v*)) of DPPH solution in ethanol, 80 µL (40% (*v*/*v*)) of buffer solution provided with the kit, and 20 µL (10% (*v*/*v*)) of sample solution. The sample solutions were prepared in ethanol or, for ethanol-insoluble compounds, in dimethyl sulfoxide (DMSO). Absorbance measurements were performed using Thermo Scientific SkanIt™ software (Ver 7.0.2). For a preliminary experiment, sample solutions were prepared at concentrations of 1, 10, 100, and 1000 µg/mL. These solutions were reacted with specific amounts of DPPH solution to determine the optimal range of IC_50_. Subsequently, the DPPH radical-scavenging rate was measured at four points within the optimal concentration range. After confirming the linearity of the scavenging rate with concentration changes and the presence of the 50% scavenging rate point on the line, a regression line was drawn, and the IC_50_ (µg/mL) was determined via interpolation. For each experiment, the IC_50_ of 6-hydroxy-2,5,7,8-tetramethyl-3,4-dihydrochromene-2-carboxylic acid (Trolox) was measured to correct for interexperimental variation. The Trolox equivalent antioxidant capacity (TEAC) was calculated using Equation (1). Furthermore, pIC_50_ (Equation (2)), which is the common logarithm of the reciprocal of IC_50_ (mol/L), was calculated. For samples whose optimal concentration range exceeded 1000 µg/mL, accurate IC_50_ measurement was not performed, as the TEAC value was expected to be extremely low.

(1) $$TEAC=\frac{{IC}_{50}\left(Trolox\right)}{{IC}_{50}\left(Sample\right)}$$

(2) $${pIC}_{50}=-\log\left({IC}_{50}\left(mol/L\right)\right)$$

### 2.3. Dataset

The SMILES of the compounds contained in the foods were obtained from FooDB in December 2024. Neutral molecules with phenolic hydroxyl groups were extracted using the fr_phenol method in RDKit (ver, 2024.09.1) [47].

PubChemQC [48], a quantum chemistry database, was used to obtain quantum chemistry calculation values. PubChemQC is a database that compiles the results of structural optimization using the PM6 method and vibrational calculations using B3LYP/6-31G(d) for compounds with molecular weights less than 1000 among the compounds reported in PubChem [49]. For the compounds registered in FooDB as of December 2024, we retrieved the SMILES, highest occupied molecular orbital energy (E_HOMO), and lowest unoccupied molecular orbital energy (E_LUMO) values, yielding a total of 4547 compounds. Additionally, quantum chemistry calculation values were similarly obtained from PubChemQC for the compounds whose DPPH activity was measured in this study (96 types) and for the compounds whose DPPH activity was measured previously using the same assay kit (169 types, 9 types) [19,33]. Hereinafter, E_HOMO and E_LUMO values obtained from PubChemQC will be referred to as E_HOMO_PubChemQC and E_LUMO_PubChemQC, respectively.

### 2.4. E_HOMO_calc and E_LUMO_calc Calculations

The calculated values reported in PubChemQC were obtained under vacuum conditions using B3LYP without considering solvent effects. Therefore, we attempted to improve calculation accuracy using B3LYP/6-31G(d)//PM6 in PubChemQC while accounting for solvent effects, with the aim of obtaining more accurate chemical values under conditions closer to the experimental system.

Molecular objects were generated from the isomeric SMILES using RDKit, followed by desalting and searching for three-dimensionally stable conformations. The ETKDG method [50] was used for conformer searches, generating 1000 conformers per compound. Each structure was optimized using the Merck Molecular Force Field (MMFF) [51], and the most stable structure obtained was used as the initial structure for quantum chemical calculations.

For the neutral molecules obtained from these structures, Gaussian16 [52] was used to perform structural optimization at B3LYP/6-31G(d), followed by vibrational analysis at M06-2X/6-311++G(d,p). The structural optimization and vibrational analyses were performed in aqueous solvents using SMD [53]. This yielded E_HOMO and E_LUMO values. Hereinafter, the E_HOMO and E_LUMO values obtained in this manner will be denoted as E_HOMO_calc and E_LUMO_calc, respectively. Furthermore, owing to the anticipated high computational cost, E_HOMO_calc and E_LUMO_calc were obtained only for the 274 compounds for which DPPH measurement data existed.

### 2.5. Machine Learning

#### 2.5.1. Calculation Descriptor

For the 274 measured compounds, only molecules for which both PubChemQC-calculated values and the calculated values were available were considered. To account for toxicity to living organisms and membrane permeability within the body [7], the dataset (measured dataset) was narrowed down to compounds with a molecular weight of less than 500 and MolLogP between 0 and 5 (247 compounds). For each compound, molecular descriptors were calculated using RDKit. Furthermore, 2D and 3D molecular descriptors were calculated using Mordred software (ver,1.2.0) [54]. For each compound, 1000 conformers were generated and optimized using MMFF. The most stable structure obtained was used to calculate the 3D molecular descriptors using Mordreds. These molecular descriptors, along with E_HOMO_PubChemQC and E_LUMO_PubChemQC, formed an initial set of 1499 molecular descriptors.

For food ingredient compounds obtained from FooDB, we filtered compounds with molecular weights less than 500 and MolLogP between 0 and 5. Using RDKit, we extracted compounds with fr_phenol ≥ 1 (2235 compounds), forming a dataset (FooDB dataset). Molecular descriptors were calculated using RDKit and Mordreds as previously described. Structures for calculating the 3D molecular descriptors were obtained using the same method as for the molecules in the measured dataset. E_HOMO_PubChemQC and E_LUMO_PubChemQC were added to these to form the initial molecular descriptors.

#### 2.5.2. Classification Model

We used the classification model to calculate the ECFP (radius = 3, bit = 2048) [55] using RDKit and used the value as an explanatory variable for the machine learning model. A binary variable (Assay) was created, assigned with a value of 0 if the IC_50_ (µg/mL) was ≥1000 µg/mL and 1 otherwise, and used as the target variable. All compounds in the measurement dataset were used for model training and evaluation. Of the measured compounds, 50% (50 compounds) registered in FooDB were used as test data and the remaining compounds (197 compounds) were used as training data. The machine learning model was implemented using the LightGBM module [44]. Using Optuna [56], a hyperparameter search was performed 30 times based on the Tree-Structured Parzen Estimator (TPE) [57] to maximize the ROC AUC [58]. The search performed a leave-one-out cross-validation (LOOCV) on the training data to determine the hyperparameters. Subsequently, LOOCV was performed on the training data using the determined hyperparameters, and the accuracy, F1 Score [59], and Matthews Correlation Coefficient (MCC) [59] were calculated to evaluate the generalization performance of the model. Furthermore, we evaluated the prediction accuracy by calculating the accuracy, F1 Score, and MCC of the test data. Subsequently, we performed an importance analysis using Shapley’s additive explanation (SHAP) [60].

#### 2.5.3. Regression Model

The pIC_50_ was calculated using Equation (2) and used as the target variable. To distinguish compounds with IC_50_ (µg/mL) values exceeding 1000 µg/mL from those with IC_50_ values ≤ 1000 µg/mL, the pIC_50_ was calculated assuming an IC_50_ of 2000 µg/mL. Boruta-Shap [61] was used to select molecular descriptors. Among the calculated molecular descriptors, those with absolute correlation coefficients exceeding 0.9 were removed. The remaining 586 molecular descriptors were input into Boruta-Shap to select the molecular descriptors.

Using RDKit on the measurement dataset, we extracted compounds (162 in total) that contained one aromatic hydroxy group and used them for model training and evaluation. Of the extracted compounds, 50% (35 compounds) registered in FooDB were used as test data and the remainder (127 compounds) were used as training data. The machine learning model was constructed using an SVM implemented in the Scikit-Learn [62] module. During training, the molecular descriptors of the training data were standardized using Scikit-Learn’s StandardScaler. Molecular descriptors of the test data were transformed accordingly. A hyperparameter search was performed 30 times using Optuna based on TPE to minimize the root mean squared error (RMSE). Similarly to the classification model, the search involved LOOCV on the training data to determine hyperparameters. Subsequently, LOOCV was performed on the training data using the determined hyperparameters, and the coefficient of determination (q^2^) and RMSE were calculated to evaluate the generalization performance of the model. Furthermore, the prediction accuracy was evaluated using the coefficient of determination (R^2^) and the RMSE of the test data. Subsequently, an importance analysis was performed using SHAP.

## 3. Results

### 3.1. Results of DPPH Assay

As the DPPH activity data previously reported by our group [19,33] were insufficient in terms of both quantity and structural diversity for machine learning analysis, we measured DPPH radical-scavenging activity in this study. Preliminary experiments on 96 compounds revealed that 43 compounds had IC_50_ values of 1000 µg/mL or less. Additional experiments were conducted on these compounds to calculate their IC_50_ and TEAC values. No additional experiments were conducted on the remaining 53 compounds because their TEAC were expected to be very small. In addition to phenols, compounds not included in FooDB were also measured. Some compounds were soluble only in DMSO and not in ethanol. Therefore, to investigate the effect of the solvent used to dissolve the samples, experiments were conducted on six compounds using samples dissolved in ethanol and samples dissolved in DMSO. We examined the effect of different solvents on TEAC; however, as all data were within the experimental error range, we considered that the data obtained for samples dissolved in ethanol and those obtained for samples dissolved in DMSO could be treated equivalently. Details are provided in the Supplementary Information. Furthermore, our previous study has shown that data obtained using the same assay kit are comparable [19]. This resulted in dataset of measurements of 274 compounds, comprising values reported previously and those obtained in the present study. To the best of our knowledge, this is the largest dataset reported by a single laboratory.

**Figure 3:**
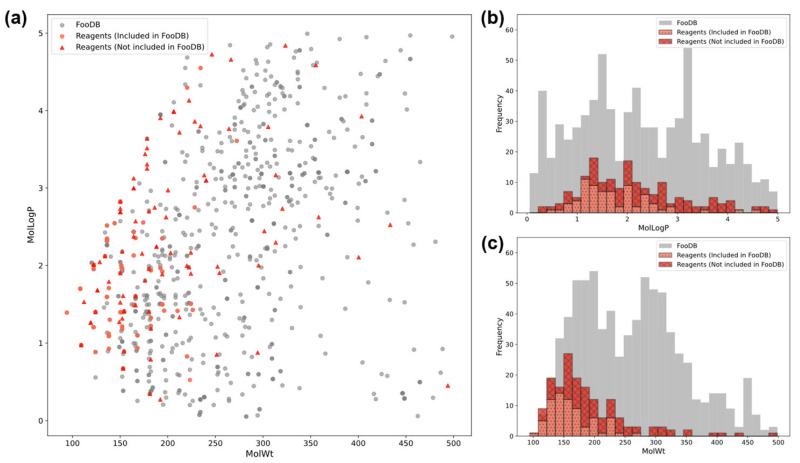
Relationship between compounds containing one phenolic hydroxyl group in the measurement dataset and compounds containing one phenolic hydroxyl group in the FooDB dataset. (**a**): Molecular weight-MolLogP plot, (**b**): MolLogP histogram, (**c**): molecular weight histogram. Histograms were stacked, with the total height representing the total number of samples.

To predict the extent to which the FooDB range would be covered by the prediction range when building a machine learning model using the measurement dataset, we investigated the difference between the compound range of the measurement dataset and that of FooDB. Figure 3a shows the MolLogP-MolWt plot for compounds with one phenolic hydroxyl group in the measurement dataset and compounds with one phenolic hydroxyl group in the FooDB dataset. Figure 3b shows that MolLogP covers a wide range of the FooDB dataset. Figure 3c shows that, with respect to molecular weight, the number of measurements for compounds with molecular weights between 300 and 500 (representing the molecular weights of most compounds in the FooDB dataset) was small, and the coverage was not as wide as that for MolLogP. However, as measurements were performed for the entire molecular weight distribution, we believe that we were able to create a broad dataset of DPPH activity measurements.

### 3.2. Comparison of Calculation Level

**Figure 4:**
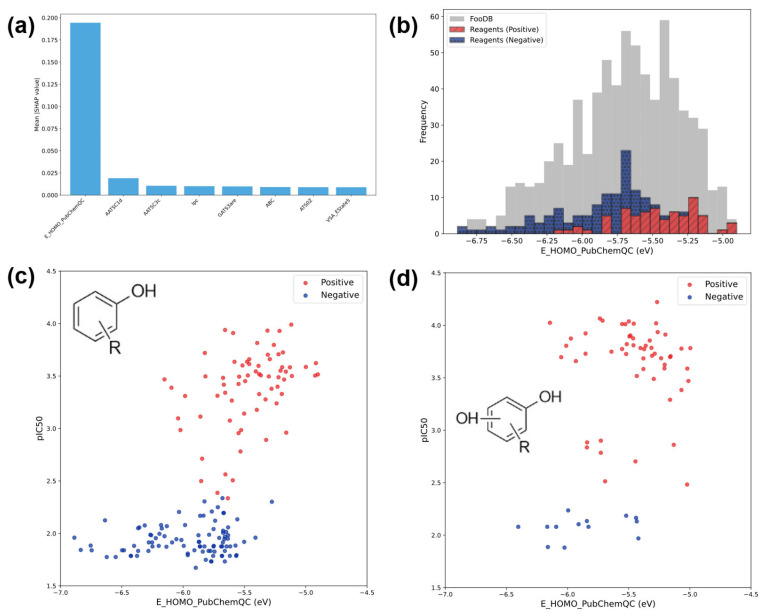
(**a**): Partial representation of feature selection and importance analysis using Boruta-Shap. (**b**): Distribution of E_HOMO_PubChemQC in the measurement dataset and FooDB dataset. (**c**): pIC_50_-E_HOMO_PubChemQC plot for compounds with one phenolic hydroxyl group in the measurement dataset. (**d**): pIC_50_-E_HOMO_PubChemQC plot for compounds with two or more phenolic hydroxyl groups in the measurement dataset. Histograms are stacked, and the total height represents the total number of samples.

Boruta-Shap was used to screen molecular descriptors and incorporate factors that significantly influenced DPPH activity into the explanatory variables input into the machine learning model. Figure 4a partially shows the results of feature selection and importance analysis using Boruta-Shap. The screening results suggest that E_HOMO is an important factor for predicting DPPH activity. The detailed results of the Boruta-Shap analysis are presented in the Supplementary Information. Figure 4b shows the distribution of E_HOMO_PubChemQC for compounds in the FooDB dataset. Compounds with higher E_HOMO values exhibited DPPH activity more frequently, whereas compounds with lower E_HOMO values were more likely to lack DPPH activity. This finding aligns with that of a previous study [63], indicating that E_HOMO influences DPPH activity, supporting the importance of E_HOMO in the reaction between DPPH and the compounds.

Next, we investigated whether the relationship between E_HOMO and pIC_50_ was affected by compound structure. Plots of pIC_50_-E_HOMO_PubChemQC for the compounds in the measurement dataset are shown in Figure 4c,d. Figure 4c shows the plot for compounds with one phenolic hydroxyl group and Figure 4d shows the plot for compounds with two or more phenolic hydroxyl groups. Figure 4c shows a narrower range of compounds with and those without DPPH activity, whereas Figure 4d shows a wider range of compounds with and those without DPPH activity. This is because compounds with two or more phenolic hydroxyl groups are more prone to subsequent reactions than those with one phenolic hydroxyl group [40] and factors other than E_HOMO are also significantly involved in the reaction. We attempted to determine a similar relationship using a higher computational level, E_HOMO_calc. There was no significant change in the plot of E_HOMO_PubChemQC vs. E_HOMO_calc, and a similar relationship was observed depending on the number of phenolic hydroxyl groups present. This finding could be attributed to the correlation between E_HOMO_PubChemQC and E_HOMO_calc (R^2^ = 0.75), which resulted in no significant difference in the overall shape of the plot. Details are provided in the Supplementary Information. Compounds with only one phenolic hydroxyl group had greater DPPH activity than compounds with two or more phenolic hydroxyl groups. Furthermore, even compounds with low E_HOMO values showed DPPH activity when the number of phenolic hydroxyl groups increased, indicating that the number of phenolic hydroxyl groups contributed to the DPPH activity in addition to E_HOMO.

### 3.3. Results of Machine Learning Analysis

#### 3.3.1. Classification Results

**Figure 5:**
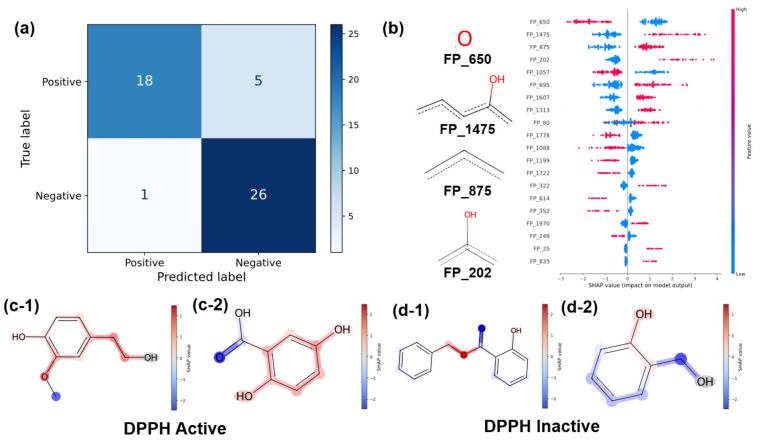
(**a**): Confusion matrix of LGBM_ECFP on test data. (**b**): SHAP (SHapley Additive exPlanations) [60] analysis of LGBM_ECFP and top four sub-structures identified as highly important. (**c-1**,**c-2**): Important structural analysis of molecules predicted as active by LGBM_ECFP. (**d-1**,**d-2**): Important structural analysis of molecules predicted as inactive by LGBM_ECFP. In panels (**c-1,c-2**,**d-1,d-2**), red indicates structural features contributing positively to the prediction of activity, whereas blue indicates features contributing negatively; darker colors correspond to larger contributions.

To achieve high accuracy and chemical interpretability of DPPH activity prediction, we first constructed a classification model (LGBM_ECFP) using ECFP as the explanatory variable. The confusion matrix for the test data composed solely of FooDB-registered compounds is shown in Figure 5a. The accuracy, F1 Score, and MCC were 0.88, 0.86, and 0.76, respectively. The LOOCV-based generalization performance evaluation of LGBM_ECFP is presented in the Supplementary Information. Furthermore, we constructed a model (LGBM_PubChemQC) in which the explanatory variable was changed from ECFP to molecular descriptors selected using Boruta-Shap. Although prediction accuracy on the test data decreased compared to that when ECFP was used as the explanatory variable (accuracy: 0.84, F1 score: 0.81, MCC: 0.68), the LOOCV generalization performance evaluation showed that it outperformed LGBM_ECFP by approximately 0.05 to 0.13 across all metrics. Furthermore, the model with a higher E_HOMO calculation level (LGBM_calc) achieved the best results for the test data (accuracy, 0.90; F1 score, 0.88; MCC, 0.80). In the LOOCV generalization performance evaluation, it also outperformed LGBM_ECFP by approximately 0.1 across all metrics. Details are provided in the Supporting Information. These results demonstrate that, although the accuracy is lower than that achieved using molecular descriptors selected using Boruta-Shap, the DPPH activity can be predicted to a certain extent using partial structures based on ECFP.

As LGBM_ECFP can classify DPPH activity to some extent based on molecular substructures, SHAP analysis was performed to identify substructures important for DPPH activity. The SHAP values represent the contribution of each feature to the model output, calculated as the difference between the prediction for a given sample and the average prediction over the background dataset. Positive and negative SHAP values indicate features that increase or decrease the predicted DPPH activity, respectively [60]. Figure 5b shows the results of the importance analysis for LGBM_ECFP and some of the partial structures predicted to contribute to the activity. Figure 5(c-1,c-2) shows some of the compounds predicted to have activity and Figure 5(d-1,d-2) shows some of the compounds predicted to lack activity. In Figure 5(c-1,c-2,d-1,d-2), the substructures that contribute positively according to the SHAP analysis are shown in red, those that contribute negatively are shown in blue, and those that contribute nothing are shown in gray. As shown in Figure 5b, the hydroxyl group of FP_202 contributes significantly and positively to the prediction of the presence of that substructure, suggesting that the model meaningfully learns. In Figure 5(c-1,c-2), the molecules predicted to be active with phenolic hydroxyl groups are shown in red. However, hydroxyl groups that are not directly bonded to the aromatic ring are not very important, suggesting that phenolic hydroxyl groups are important for DPPH activity and that phenols tend to act as antioxidants. The ortho-methoxy groups are shown in red, indicating that they contributed significantly. This observation supports the findings of a previous study [64] showing that ortho-methoxy groups affect activity. In contrast, Figure 5(d-1,d-2) shows that the molecules predicted to be inactive have carbon atoms on the benzene ring without substituents, which are displayed in blue. This finding suggests that the presence of substituents tends to increase the DPPH activity. Furthermore, the oxygen of the carboxyl group contributed negatively to this prediction. This finding is in agreement with the results of a previous study [64]. Thus, the important substructures obtained by analyzing LGBM_ECFP can be explained to some extent by previously reported findings, suggesting that the machine learning model performs meaningful learning and provides highly accurate predictions, while also indicating the possibility of obtaining new findings.

#### 3.3.2. Regression Results

**Figure 6:**
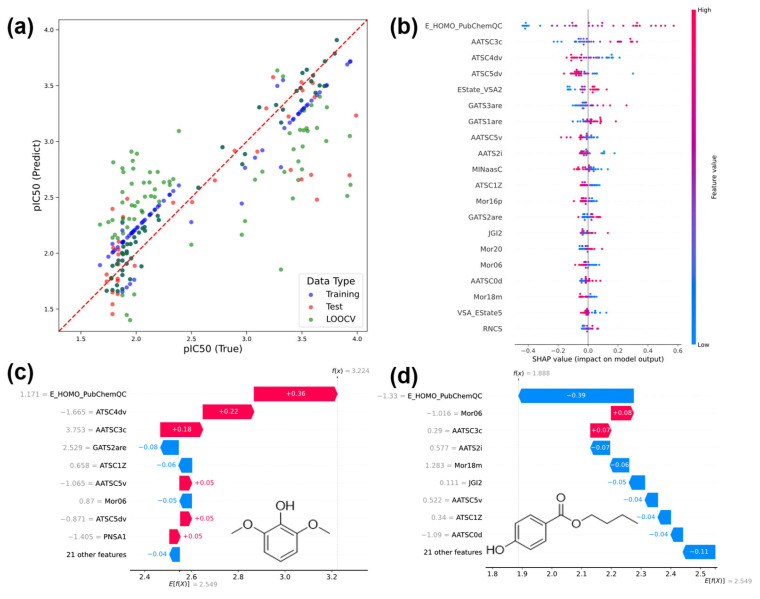
(**a**): YY plot of SVR_PubChemQC, (**b**): SHAP-summary-plot of SVM_PubChemQC, (**c**): SHAP analysis of 2,6-dimethoxyphenol, (**d**): SHAP analysis of 4-hydroxybenzoic acid butyl ester.

The classification model can predict the presence or absence of DPPH activity; however, predicting antioxidant capacity of a compound requires not only determining whether activity is present, but also its strength. Therefore, we constructed a regression model that can predict the strength of activity as a continuous value. Initially, we applied the regression model to all molecules, but did not obtain satisfactory prediction accuracy. The data provided in Figure 4c,d suggest that the number of phenolic hydroxyl groups affects the relationship between E_HOMO and pIC_50_; so, we predicted molecules with only one phenolic hydroxyl group. Figure 6a shows the YY plot of the model (SVM_PubChemQC) trained with features selected using Boruta-Shap. The R^2^_test_ was 0.70, RMSE_test_ was 0.44, q^2^ was 0.61, and RMSE_validation_ was 0.46, satisfying the Golbraikh–Tropsha criteria [65]. To improve the accuracy of the machine learning model, we constructed a new model (SVM_calc) by replacing E_HOMO_PubChemQC with E_HOMO_calc, which was calculated at a higher level. SVM_calc had an R^2^_test_ of 0.71, an RMSE_test_ of 0.44, a q^2^ of 0.59, and an RMSE_validation_ of 0.48. The model details are provided in the Supplementary Information. The potential reason why the prediction accuracy did not change significantly despite increasing the E_HOMO calculation level is that E_HOMO_PubChemQC and E_HOMO_calc were correlated, and the relative strength of E_HOMO did not change significantly depending on the calculation level. This finding suggests that DPPH activity can be predicted to a certain extent for molecules with only one phenolic hydroxyl group.

To investigate the factors influencing DPPH activity, SHAP analysis was performed using SVM_PubChemQC. In this study, we examined both factors effectively across the entire dataset and those based on activity strength. The SHAP summary plot of SVM_PubChemQC is presented in Figure 6b. The SHAP analysis results for 2,6-dimethoxyphenol, a compound with pIC_50_ ≥ 3, are shown in Figure 6c. The SHAP analysis results for 4-hydroxybenzoic acid butyl, a compound with pIC_50_ < 2, are shown in Figure 6d. Figure 6b shows that E_HOMO contributed significantly to the overall prediction. This finding supports that of prior research [63], indicating a large contribution from E_HOMO, as mentioned earlier. Comparing Figure 6c,d, E_HOMO contributes positively to the prediction in Figure 6c but negatively to that in Figure 6d. This observation indicates that within the measured dataset, 2,6-dimethoxyphenol had a relatively high E_HOMO, whereas 4-hydroxybenzoic acid butyl ester had a low E_HOMO. Here, we considered the reaction mechanism between DPPH and these compounds. Three mechanisms have been proposed for the DPPH–compound reactions: HAT, ET, and SPLET [66]. Among these, the ET mechanism is thought to be related to the IP. A strong correlation between IP and E_HOMO has been reported. Furthermore, as the ET mechanism involves a cationization reaction in which electrons are abstracted, it is considered to be within the applicability range of Koopman’s theorem [67]. Consequently, the predictions using SVM_PubChemQC indicated a significant contribution from E_HOMO. This finding suggests that the ET mechanism, in which IP serves as a considerable driving force, is likely to occur in the reaction between DPPH and the compound, consistent with the findings of previous research [19,68].

### 3.4. Prediction of FooDB Compounds

**Figure 7:**
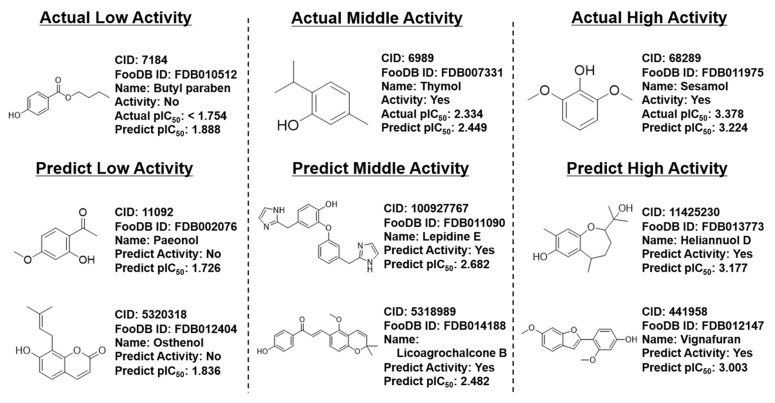
Some of the food component compounds whose DPPH activity was predicted.

As the DPPH activity of foods can be roughly calculated as the sum of the DPPH activities of the antioxidants in the food, regression prediction of the DPPH activity of food constituents is considered important. First, classification prediction was performed using LGBM_ECFP for 2235 phenols included in FooDB with a molecular weight of less than 500 and a MolLogP of 0 or more and less than 5. Among these, 1225 phenols were predicted to have DPPH activity, while 1010 phenols were predicted to not have DPPH activity. Next, regression prediction was performed for 753 phenols with one phenolic hydroxyl group, for which E_HOMO was obtained from PubChemQC. Among them, 148 compounds were predicted to exhibit high activity, with pIC_50_ exceeding 3. Compounds predicted to have high pIC_50_ values and low pIC_50_ values, and no DPPH activity are shown in Figure 7. Details of the classification and regression predictions are presented in the Supplementary Information. By predicting the continuous value of pIC_50_ for food component compounds with one phenolic hydroxyl group, it is possible to predict the presence and strength of antioxidant activity, which has been challenging until now. We believe that quantitative prediction of antioxidant activity enables efficient exploration of the chemical space for expensive reagents that are difficult to experiment with and food component compounds that are difficult to extract. Furthermore, we have laid the foundation for the quantitative prediction of the antioxidant activity of food components.

### 3.5. Limitations and Directions for Future Work

Regarding the evaluation metrics used for the classification models, each metric has inherent limitations. Accuracy is sensitive to class imbalance and may overestimate performance for the majority class. The F1 score, defined as the harmonic mean of precision and recall, does not consider true negatives and therefore does not fully reflect the model’s ability to correctly exclude inactive compounds. Although the MCC incorporates all elements of the confusion matrix and is relatively robust to class imbalance, its value can become unstable when applied to relatively small datasets, such as those evaluated using LOOCV, and should therefore be interpreted with caution. The regression prediction model constructed in this study showed high adaptability for compounds containing only one phenolic hydroxyl group; however, its predictive accuracy could be improved by incorporating more complex descriptors, such as bond dissociation energy and acidity constant, as well as by expanding the dataset. Furthermore, the model cannot be applied to compounds with two or more phenolic hydroxyl groups, which are major contributors to antioxidant capacity. Therefore, to quantify the antioxidant capacities of foods more comprehensively, it is essential to include compounds with multiple hydroxyl groups in the model. While the descriptors and non-deep learning models currently in use may not be able to fully reflect the DPPH activity of these compounds, the introduction of machine learning methods with excellent nonlinear feature extraction capabilities, such as deep learning, is expected to enable more accurate handling of a wider variety of phenols for accurate prediction, albeit at the cost of interpretability. The results of this study lay the foundation for this and, in the future, it may be possible to develop a platform for comprehensively predicting and comparing the antioxidant capacities of a wide range of phenolic compounds in foods. Previous studies (e.g., [15]) have shown that the sum of the antioxidant capacities of food components is almost equal to the antioxidant capacity of the food itself. We believe that by predicting the antioxidant capacity of food components, it is possible to predict the antioxidant capacity of the food itself.

## 4. Conclusions

In this study, we established a foundation for quantitatively predicting the antioxidant capacity of numerous phenolic compounds, which affect the antioxidant capacity of foods. We measured DPPH activity of 96 compounds present in food components and added the measurements to the current dataset. Including previously reported data, we created a dataset of 274 compounds, which, to our knowledge, is the largest single-laboratory dataset. Quantum chemical calculations were performed for these compounds and the calculated values were compared with the TEAC and pIC_50_ values. In addition, a DPPH activity prediction model was constructed using machine learning to predict the DPPH activity more accurately. This prediction model performance was satisfactory, and the model satisfied the Golbraikh–Tropsha criteria. Furthermore, by obtaining quantum chemical calculation values from PubChemQC, a model constructed for other food components was adapted. To our knowledge, this is the largest study to quantitatively predict the DPPH activity of food components. Each food contains numerous components, and they need to be extracted individually to perform quantitative measurements of antioxidant capacity, making prediction challenging. This study contributes to the quantitative elucidation of the antioxidant capacities of foods containing various components.

## Data Availability

The original contributions presented in this study are included in the article/Supplementary Materials. Further inquiries can be directed to the corresponding author(s).

## Supplementary Materials

The following supporting information can be downloaded at: https://www.mdpi.com/article/10.3390/cimb48010012/s1.

## Abbreviations

The following abbreviations are used in this manuscript:

DPPH	2,2-Diphenyl-1-Picrylhydrazyl
ROS	Reactive Oxygen Species
SAR	Structure–Activity Relationship
IP	Ionization Potential
NNs	Neural Networks
DMSO	Dimethyl Sulfoxide
Trolox	6-Hydroxy-2,5,7,8-Tetramethyl-3,4-Dihydrochromene-2-Carboxylic Acid
TEAC	Trolox Equivalent Antioxidant Capacity
E_HOMO	Highest Occupied Molecular Orbital Energy
E_LUMO	Lowest Unoccupied Molecular Orbital Energy
MMFF	Merck Molecular Force Field
TPE	Tree-Structured Parzen Estimator
LOOCV	Leave-One-Out Cross-Validation
MCC	Matthews Correlation Coefficient
SHAP	Shapley’s Additive Explanation
SVM	Support Vector Machine
RMSE	Root Mean Squared Error
