# Supplementary material for: DPPH Measurement for Phenols and Prediction of Antioxidant Activity of Phenolic Compounds in Food
Source: Curr Issues Mol Biol. 2025 Dec 23;48(1):12. doi: 10.3390/cimb48010012 (PMC12840554; doi:10.3390/cimb48010012)
Supplement: Supplementary file 1 [file cimb-48-00012-s001.zip › SI_ver10.pdf]

Supporting Information

DPPH Measurement for Phenols and Prediction of Antioxidant Activity of Phenolic Compounds in Food

Riku Kato, Chihiro Tada, Moeka Yamauchi, Yuuto Matsumoto, and Hiroaki Gotoh

Table of Contents

0. Introduction - P.S3
1. Changes in DPPH Activity Due to Differences in Solvents - P.S4
2. Results of Feature Selection for the Boruta-Shap Regression Model - P.S5
3. Relationship between E_HOMO Calculation Levels and DPPH Activity - P.S11
4. Classification Model Details
4.1. Hyperparameters Determined by Optuna - P.S12
4.2. Comparison of LGBM_ECFP, LGBM_PubChemQC, and LGBM_calc - P.S12
5. Regression Model Details
5.1. Hyperparameters Determined by Optuna - P.S15
5.2. Comparison of SVM_PubChemQC and SVM_calc - P.S15
6. About Synthetic Products - P.S17

0. Introduction

In Excel (SI.xlsx), the measured values of the compounds used in the experiment and the results of quantum chemical calculations are shown in Sheet 1 (Experimental Data), the molecules registered in FooDB that were predicted by this classification model and the prediction results of LGBM_ECFP are shown in Sheet 2 (Predict_Classification), and the molecules that were predicted by the regression model, the molecular descriptors used, and the prediction results of SVM_PubChemQC are shown in Sheet 3 (Predict_Regression).

1. Changes in DPPH Activity Due to Differences in Solvents

To investigate the effect of solvent on DPPH activity, we investigated the difference in DPPH activity when six compounds were dissolved in ethanol and DMSO. While there was a tendency for activity to decrease slightly when dissolved in DMSO, the difference in pIC_{50} was less than 0.2, which is considered within the experimental error range. However, the low activity of the DMSO-dissolved samples suggests that there is at least some solvent effect. The measured values are listed in Table 1, and a plot of the measured values is shown in Figure 1.

Table S1. Changes in DPPH activity with different solvents

Compound	pIC_{50} (EtOH)	pIC_{50} (DMSO)
Pyrogallol	4.010	3.903
Gallic acid	4.010	3.961
Sesamol	3.582	3.470
2-tert-Butyl-4-ethylphenol	2.984	2.874
N-Carbobenzoxy-4-hydroxy-D-2-phenylglycine	2.985	2.873
3',4'-Dihydroxyacetophenone	3.873	3.935

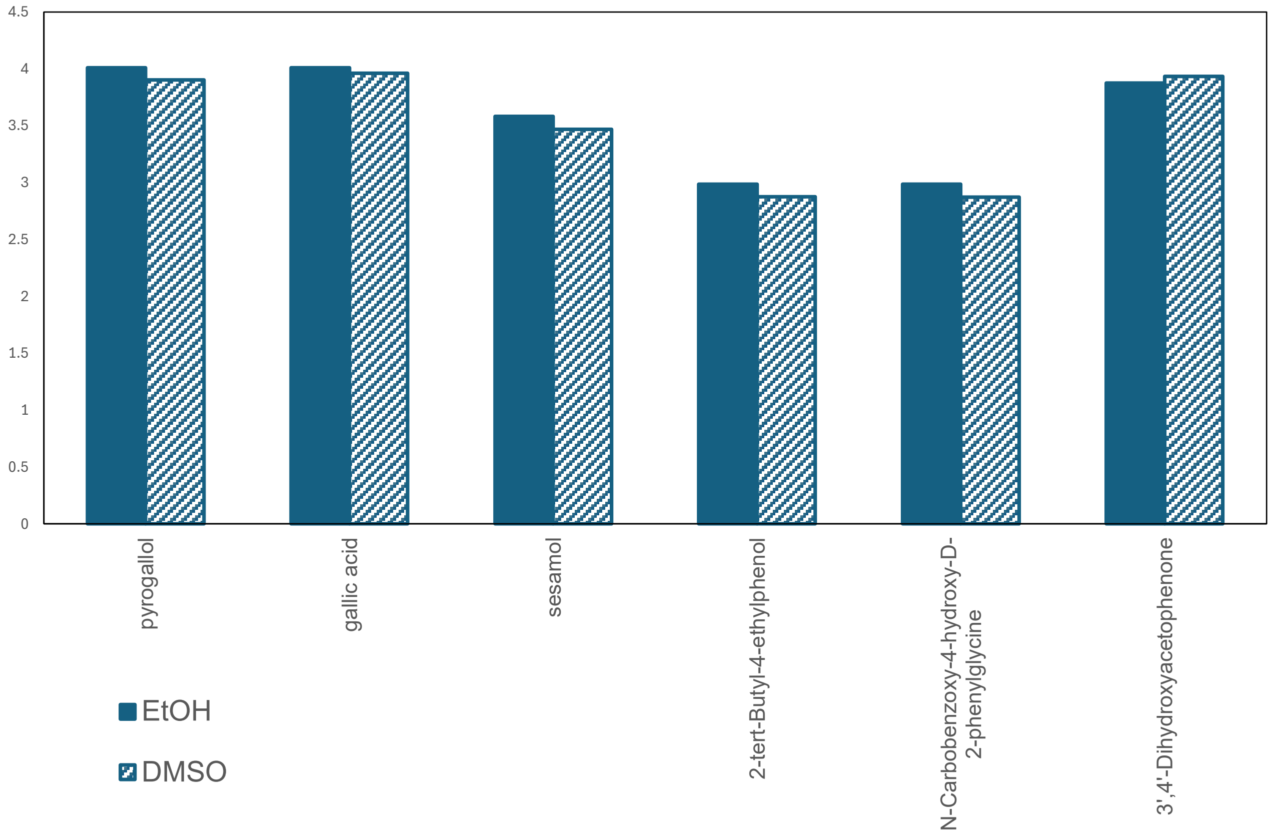

Figure S1. Changes in DPPH activity with different solvents.

2. Results of Feature Selection for the Boruta-Shap Regression Model

The percentile parameter of Boruta-Shap is the threshold used to determine whether a feature is important, and it is determined by the percentile of the shadow feature distribution it exceeds. The

initial value of 100 may be too strict, resulting in very few features being selected. In this study, when performing regression prediction, only a small number of features were selected, so it was set to 95, which is generally considered appropriate. The results of feature selection and importance analysis using Boruta-Shap are shown in Figure SI2, along with a heatmap of the selected features. Details of each descriptor are provided below with reference to references [1,2]. The values in parentheses indicate the model that uses that molecular descriptor as a feature.

〇ATS

In a molecular graph, the autocorrelation of atomic property values is calculated according to the number of bonds between atoms (topological distance). The numbers represent the topological distance, and the calculation is performed using the atomic property indicated by the alphabet following it. Examples of atomic properties are shown in Table S2.

Used in this study

・ATS0Z (SVM_PubChemQC, SVM_calc)

〇AATS

In a molecular graph, the autocorrelation (ATS) of atomic property values is calculated according to the number of bonds between atoms (topological distance), and then divided (averaged) by the number of atom pairs. The numbers represent the topological distance, and the calculation is performed using the atomic property indicated by the alphabet following it. Examples of atomic properties are shown in Table SI2.

Used in this study

・AATS2i (SVM_PubChemQC)

・AATS3i (SVM_calc)

・AATS5dv (SVM_PubChemQC, SVM_calc)

〇ATSC

It is calculated in the same way as ATS, but instead of using the atomic property values themselves, it replaces them with the deviation from the average. As with AATS, the numbers represent the topological distance, and the calculation is performed using the atomic property represented by the alphabet following it. Examples of atomic properties are shown in Table SI2.

Used in this study

・ATSC1dv (SVM_calc)

・ATSC1Z (SVM_PubChemQC)

・ATSC4c (LGBM_PubChemQC, LGBM_calc)

・ATSC4dv (LGBM_PubChemQC, LGBM_calc, SVM_PubChemQC)

・ATSC5dv (SVM_PubChemQC)

・ATSC6dv (SVM_calc)

・ATSC6i (SVM_calc)

・ATSC7dv (LGBM_PubChemQC, LGBM_calc, SVM_calc)
・ATSC8are (SVM_calc)

○AATSC
・AATSC1d (SVM_PubChemQC)
It is calculated in the same way as ATS, but instead of using the atomic property values themselves, it replaces them with the deviation from the average and calculates the result (ATSC), which is then divided by the number of atom pairs (averaged). As with AATS, the numbers represent the topological distance, and the calculation is performed using the atomic property indicated by the alphabet following it. Examples of atomic properties are shown in Table SI2.

Used in this study
・AATSC0d (SVM_PubChemQC)
・AATSC1d (SVM_PubChemQC)
・AATSC3c (LGBM_PubChemQC, LGBM_calc, SVM_PubChemQC, SVM_calc)
・AATSC4c (LGBM_PubChemQC, LGBM_calc)
・AATSC5v (SVM_PubChemQC)

○GATS
This shows how different a certain atomic property is in a molecular graph at its topological distance. The number represents the topological distance, calculated using the atomic property indicated by the alphabet following it. Examples of atomic properties are shown in Table SI2.

Uses in this study
・GATS1are (LGBM_PubChemQC, LGBM_calc, SVM_PubChemQC)
・GATS1dv (LGBM_PubChemQC, LGBM_calc)
・GATS2are (SVM_PubChemQC)
・GATS3are (SVM_PubChemQC)
・GATS4dv (LGBM_calc)

○Mor
This represents the intensity of an electron diffraction pattern in three-dimensional space. The numbers represent scattering factors; larger numbers reflect more local information, whereas smaller numbers reflect the entire molecule. Calculations are based on the atomic properties indicated by the alphabet following the number. Examples of atomic properties are shown in Table SI2.

Used in this study
・Mor06 (SVM_PubChemQC)
・Mor06p (SVM_PubChemQC)
・Mor08p (SVM_calc)

・Mor09m (SVM_calc)
・Mor13 (LGBM_PubChemQC, LGBM_calc)
・Mor13m (LGBM_PubChemQC, LGBM_calc)
・Mor15 (LGBM_PubChemQC, LGBM_calc)
・Mor16p (LGBM_PubChemQC, LGBM_calc, SVM_PubChemQC)
・Mor18m (LGBM_PubChemQC, LGBM_calc, SVM_PubChemQC)
・Mor19 (LGBM_calc)
・Mor19m (LGBM_PubChemQC, LGBM_calc)
・Mor20 (SVM_PubChemQC, SVM_calc)
・Mor20m (LGBM_PubChemQC, LGBM_calc, SVM_calc)
・Mor22 (SVM_calc)
・Mor27 (SVM_calc)
・Mor28 (SVM_calc)

〇Other
・ABC (SVM_PubChemQC, SVM_calc)
This indicates the symmetry of a molecule based on its three-dimensional structure. The smaller the value, the higher the symmetry; the larger the value, the higher the asymmetry.

・BalabanJ (SVM_PubChemQC, SVM_calc)
Structural complexity measured by the differences between atoms in the undirected graph of a molecule. Higher values indicate a more cyclic and symmetrical structure, whereas lower values indicate a more linear structure.

・BCUT2D_LOGPHI (LGBM_PubChemQC, LGBM_calc, SVM_calc)
Eigenvalues of the Burden matrix weighted by logP for each atom based on the molecular graph. The larger the value, the more concentrated are the hydrophobic parts, and the smaller the value, the more dispersed are the hydrophobic parts.

・E_HOMO (LGBM_PubChemQC, LGBM_calc, SVM_PubChemQC, SVM_calc)
Highest Occupied Molecular Orbital Energy

・E_LUMO (LGBM_PubChemQC, LGBM_calc)
Lowest Unoccupied Molecular Orbital Energy

・Estate_VSA2 (SVM_PubChemQC, SVM_calc)
The molecules are divided into groups according to their molecular area, and the total E-State value for each group is shown. The groups are indicated by the numbers at the end, and there are 10 groups.

・Ipc (SVM_PubChemQC, SVM_calc)
This is a calculation of the structural diversity and symmetry of a molecule. The larger the value, the more atoms with different numbers of bonds in the molecule, and the smaller the value, the more atoms with the same number of bonds.

・JGI2 (SVM_PubChemQC, SVM_calc)
This represents the charge correlation between atoms across two bonds using the Gasteiger Charge. A larger value indicates a stronger inductive effect, whereas a smaller value indicates a stronger non-polarity of the molecule.

・JGI3 (SVM_calc)
This represents the charge correlation between atoms across three bonds using the Gasteiger Charge. A larger value indicates a stronger inductive effect, whereas a smaller value indicates a stronger non-polarity of the molecule.

・MaxAbsEStateIndex (LGBM_calc)
The maximum absolute value of the E-State of the atom in the most extreme electronic environment within a molecule. The larger the value, the stronger is the asymmetry of the electron distribution.

・MaxAbsPartialCharge (LGBM_PubChemQC, LGBM_calc)
The molecule with the largest absolute partial charge determined using Gasteiger charges.

・MaxPartialCharge (LGBM_PubChemQC, LGBM_calc)
The molecule with the largest partial charge determined using Gasteiger charges.

・MinAbsPartialCharge (LGBM_PubChemQC, LGBM_calc)
The molecule with the smallest absolute partial charge determined using Gasteiger charges.

・MlNaasC (SVM_PubChemQC, SVM_calc)
This is the sum of the absolute values of the Gasteiger charges of the atoms surrounding each carbon atom calculated for the entire molecule. The higher the value, the more polar and electronically heterogeneous is the molecule.

・MOMI-X (SVM_PubChemQC, SVM_calc)
This represents how much the mass is spread along the x-axis in three-dimensional space. A larger value indicates a more spread-out structure, whereas a smaller value indicates a long, linear molecule.

・PNSA1 (SVM_PubChemQC, SVM_calc)
The sum of the surface area of the negatively charged parts of the molecular surface of the three-dimensional structure determined using the Gasteiger charge.

・RNCS (SVM_PubChemQC, SVM_calc)

This represents the ratio of bonds held by secondary carbon atoms to the total bonds in the molecule. A larger value indicates more branches within the molecule.

・SaacC (SVM_PubChemQC)

The sum of all E-State values of only carbon atoms in a molecule. The larger the absolute value, the greater the bias in electron distribution. A positive value indicates that there are more carbon atoms adjacent to electron-withdrawing atoms, and a negative value indicates that there are more carbon atoms adjacent to electron-donating atoms.

・SlogP_VSA11 (LGBM_PubChemQC, LGBM_calc)

The surface area of a molecule occupied by atoms with a logP greater than 1.05.

・SMR_VSA9 (LGBM_PubChemQC, LGBM_calc)

The surface area of a molecule that is occupied by atoms with an SMR (which indicates the hydrophobicity or polarizability of a molecule) of more than 0.9. The larger the value, the more hydrophobic carbon or halogen, and the lower the value, the more polar groups and fewer hydrophobic surfaces.

・VSA_Estate5 (LGBM_calc、SVM_PubChemQC、SVM_calc)

The atoms are grouped according to their E-State values, and the total molecular surface area of each group is calculated.

Table S2. Atomic property examples.

symbol	meaning
Z	atomic number
i	ionization potential
dv	atomic van der Waals volume (derived value)
c	partial charge
are	atomic relative electronegativity
d	Sanderson atomic dipole polarizability
v	van der Waals volume
p	polarizability
m	mean atomic mass

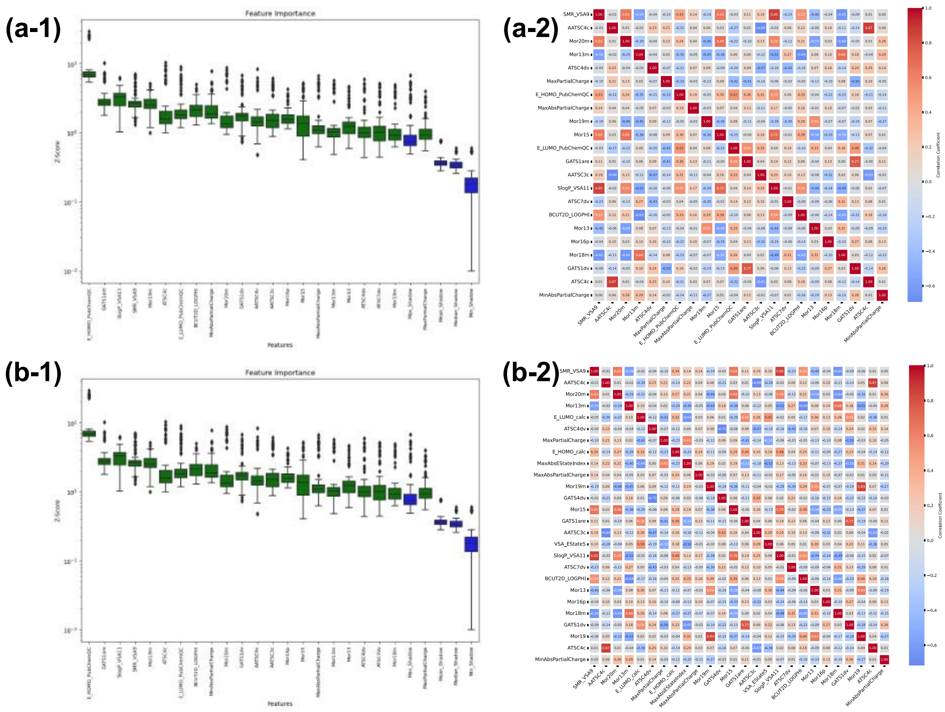

Figure S2. Boruta-Shap results and heatmap for classification models. (a): LGBM_PubChemQC (b): LGBM_calc.

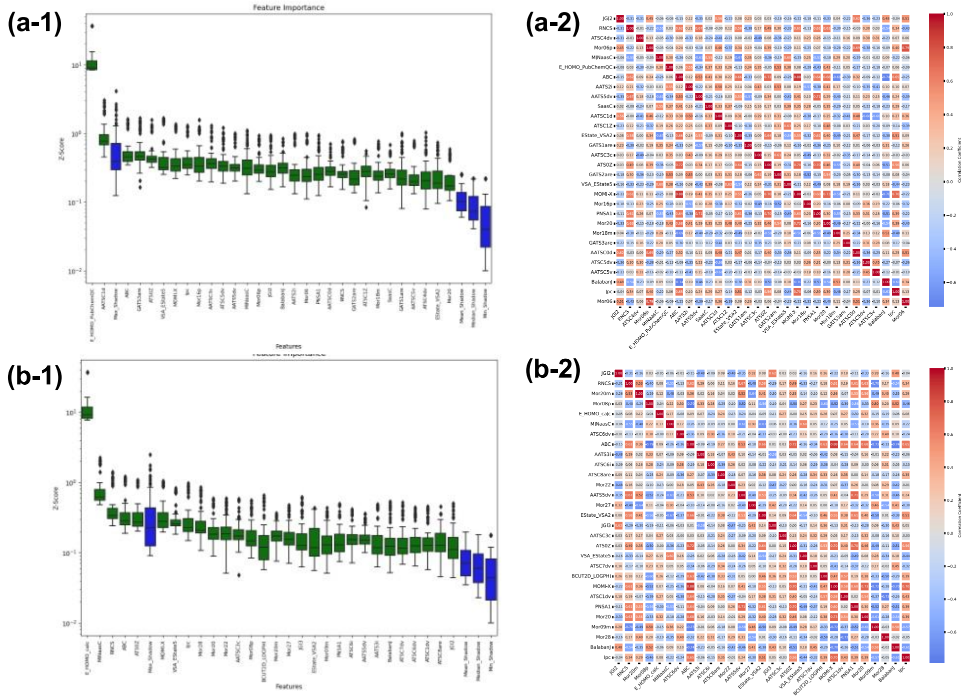

Figure S3. Boruta-Shap results and heatmap for regression models. (a): SVM_PubChemQC (b): SVM_calc.

3. Relationship between E_HOMO Calculation Levels and DPPH Activity

The relationship between pIC_{50} and E_HOMO_calc is shown in Figure SI4(a) for compounds with one phenolic hydroxy group and in Figure SI3(b) for compounds with two or more phenolic hydroxy groups. The outline does not change significantly compared to Figures 4(c) and (d) in the main text. In this study, we constructed a machine learning model using E_HOMO_PubChemQC, for which data are more readily available, allowing for easy predictions even for unmeasured molecules.

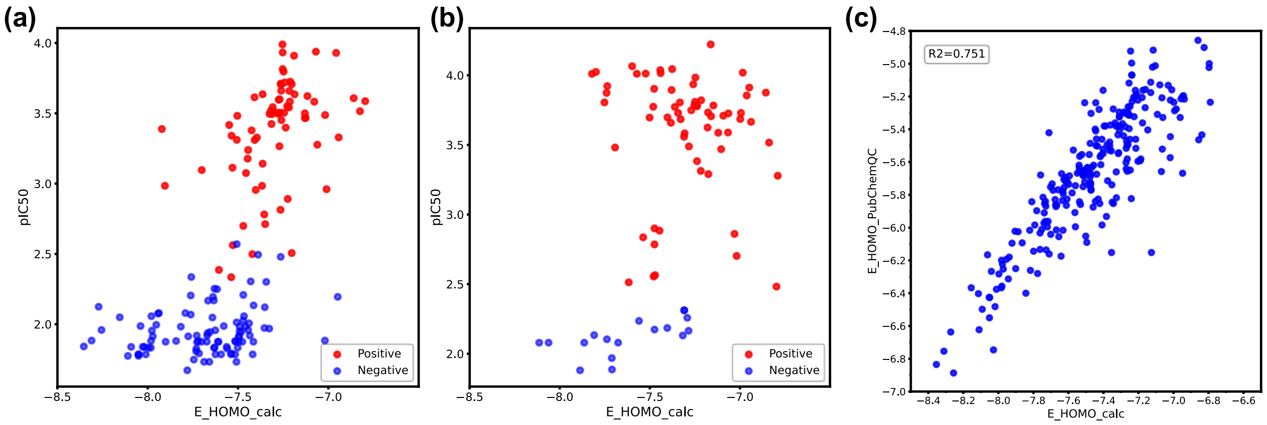

Figure S4. (a): pIC_{50}-E_HOMO_calc plot for compounds with one phenolic hydroxy group in the measurement dataset.
(b): pIC_{50}-E_HOMO_calc plot for compounds with two or more phenolic hydroxy groups in the measurement dataset.
(c): E_HOMO_PubChemQC-E_HOMO_calc plot.

4. Classification Model Details

4-1. Hyperparameters Determined by Optuna

In this study, we investigated the radius of ECFP. The model constructed using ECFP is called LGBM_ECFP, and the value in parentheses indicates the radius of ECFP. In addition to ECFP, models were constructed for descriptors selected by Boruta-Shap, and the accuracy of the models was compared. The models constructed using features selected by Boruta-Shap are called LGBM_PubChemQC and LGBM_calc. Each model uses a different E_HOMO calculation level: LGBM_PubChemQC uses E_HOMO_PubChemQC and LGBM_calc uses E_HOMO_calc. When determining the hyperparameters, Optuna was used to perform 50 searches based on TPE, and the results were determined to maximize the ROC_AUC. The hyperparameters for each model are shown in Table SI3.

Table S3. Classification model hyperparameters.

model	n_estimator	max_depth	learning_rate	num_leaves	min_child_samples	subsamples	colsamplre_bytree
LGBM_ECFP(2)	147	4	0.267	14	30	0.66	0.53
LGBM_ECFP(3)	124	6	0.170	11	12	0.80	0.85
LGBM_PubChemQC	198	7	0.006	23	8	0.87	0.85
LGBM_calc	36	9	0.095	8	19	0.50	0.99

4-2. Comparison of LGBM_ECFP, LGBM_PubChemQC, and LGBM_calc

The confusion matrix for the evaluation of LGBM_ECFP(2) and LGBM_ECFP(3) is shown in Figure SI4. The top four substructures determined to be important by Shap analysis are also shown.

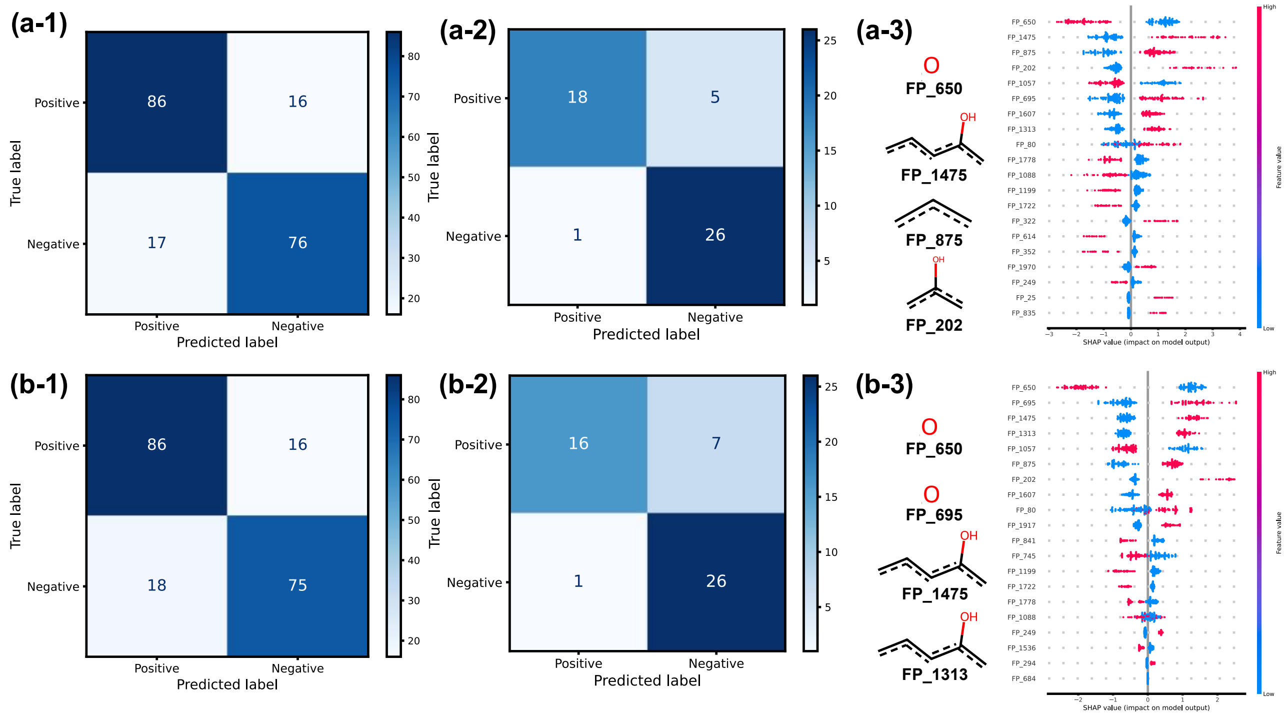

Figure S5. Confusion matrix of LOOCV and test data and Shap analysis results of the model using ECFP.

(a): LGBM_ECFP(2).

(b): LGBM_ECFP(3).

In addition, the results of LOOCV and Shap analysis of the confusion matrix and model for the test data are shown in Figure SI6 as an evaluation of LGBM_PubChemQC and LGBM_calc.

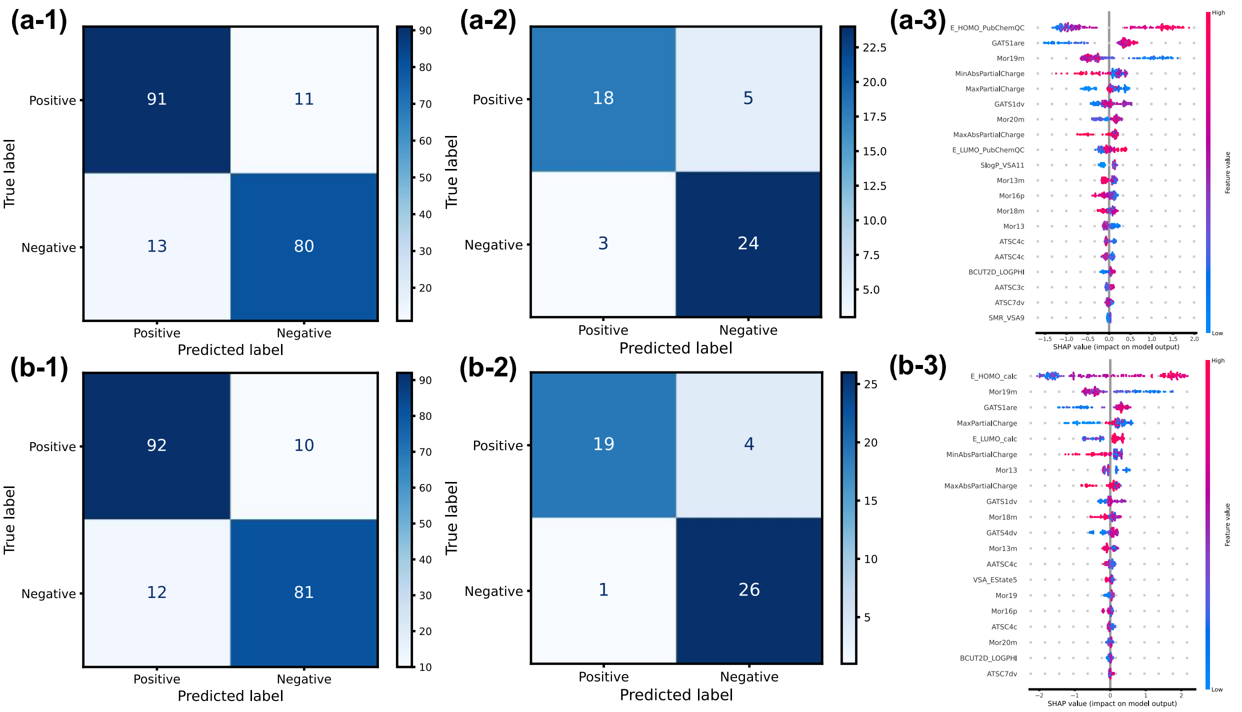

Figure S6. Confusion matrix of LOOCV and test data and Shap analysis results of the model using descriptors.
(a): LGBM_PubChemQC.
(b): LGBM_calc.

The evaluation values of all models are shown in Table SI4, and a comparison is shown in a bar graph in Figure SI7. LGBM_calc was the best model, but LGBM_ECFP(3) also showed good performance, coming in second. Considering the ease of calculation of ECFP and ease of interpretation, we used LGBM_ECFP(3) in this study.

Table S4. Evaluation values of all classification models.

model	Accuracy_LOOCV	Accuracy_test	F1 Score_LOOCV	F1 Score_test	MCC_LOOCV	MCC_test
LGBM_ECFP(2)	0.83	0.84	0.84	0.80	0.65	0.69
LGBM_ECFP(3)	0.83	0.88	0.83	0.86	0.66	0.76
LGBM_PubChemQC	0.88	0.84	0.88	0.81	0.75	0.68
LGBM_calc	0.89	0.90	0.89	0.88	0.77	0.80

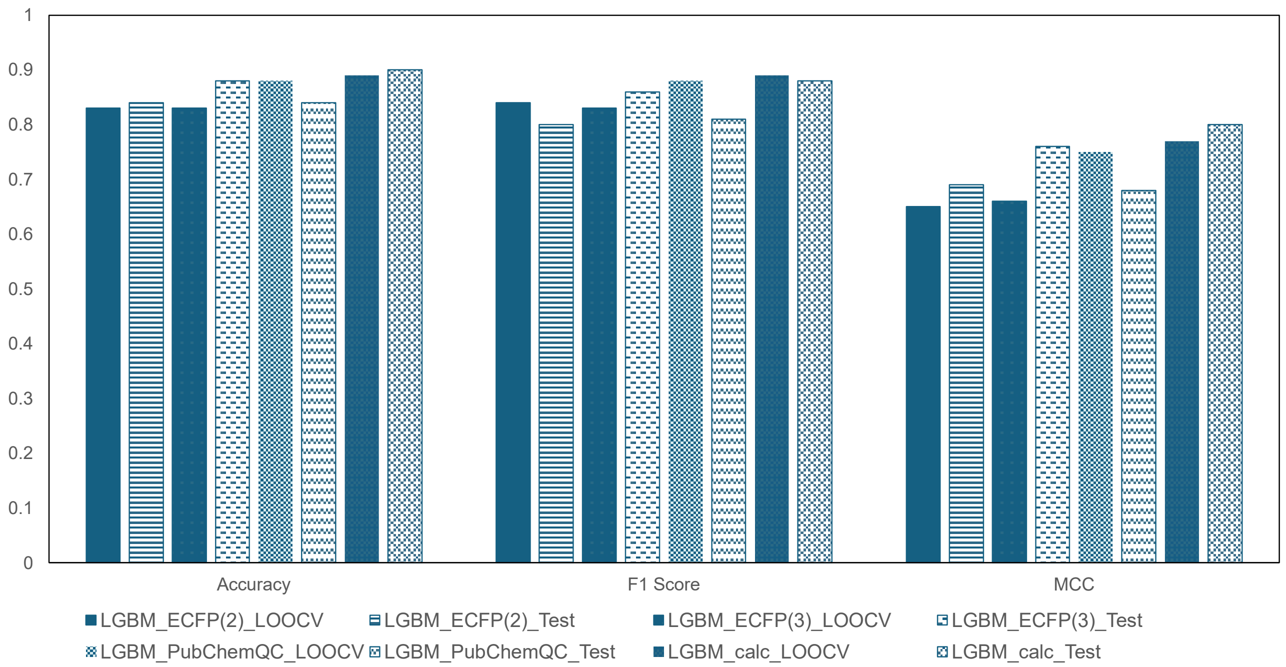

Figure S7. Comparison of evaluation values of the classification models.

5. Regression Model Details

5-1. Hyperparameters Determined by Optuna

In this study, the models constructed using the features selected by Boruta-Shap are called SVM_PubChemQC and SVM_calc. Each model uses a different E_HOMO calculation level: SVM_PubChemQC uses E_HOMO_PubChemQC and SVM_calc uses E_HOMO_calc. Optuna was used to perform 50 searches based on TPE to determine the hyperparameters, and the results were determined to maximize the ROC_AUC. The hyperparameters determined for each model are shown in Table SI5.

Table S5. Regression model hyperparameters.

model	C	epsilon	gamma
SVM_PubChemQC	26.07	0.14	auto
SVM_calc	30.43	0.20	auto

5-2. Comparison of SVM_PubChemQC and SVM_calc

The evaluation scores for SVM_PubChemQC and SVM_calc are shown in Table SI6, and the results of the SVM_calc YY plot and Shap analysis are shown in Figure SI8. For the results of the SVM_PubChemQC YY plot and Shap analysis, please refer to Figure 5 in the main text. These results show that E_HOMO contributes significantly to DPPH activity; however, improving the calculation level did not significantly improve the predictions. We believe this is because there are other factors besides E_HOMO that contribute significantly to DPPH activity.

Table S6. Comparison of the regression models.

model	R^2_test	RMSE_test	q^2	RMSE_LOOCV
SVM_PubChemQC	0.70	0.44	0.61	0.46
SVM_calc	0.71	0.44	0.59	0.48

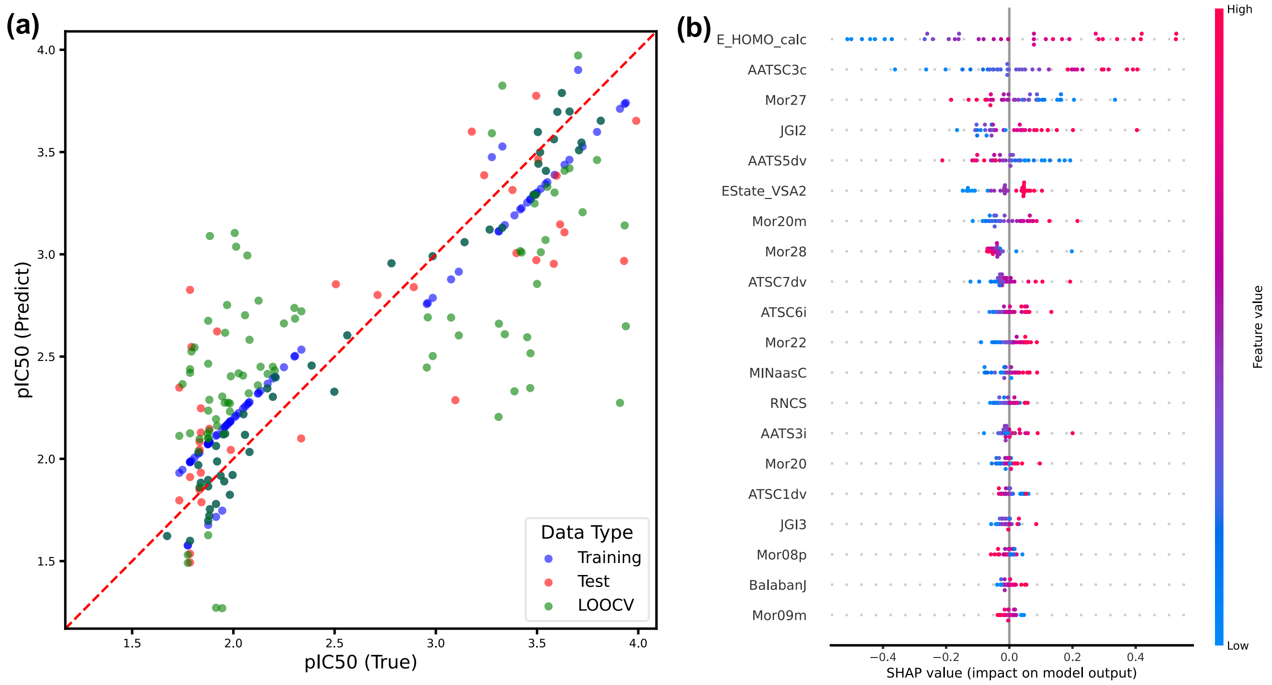

Figure S8. (a): YY plot of SVM_calc (b): SVM_calc Shap analysis results.

6. About Synthetic Products

Among the compounds for which DPPH measurements were newly performed in this study, eight were synthetic. All of these compounds were synthesized based on literature previous study [3].

References

1. RDKit. Available online: https://www.rdkit.org/ (accessed on 29 October 2025). .
2. Moriwaki, H.; Tian, Y.S.; Kawashita, N.; Takagi, T. Mordred: A molecular descriptor calculator. J. Cheminform. 2018, 10, 4. https://doi.org/10.1186/S13321-018-0258-Y.
3. Nishii, T.; Ichizawa, K.; Nagano, H.; Mukai, H.; Sakaguchi, D.; Gotoh, H. Predicting substrate reactivity in oxidative homocoupling of phenols using positive and unlabeled machine learning. ACS Omega 2025, 10, 49805–49815. https://doi.org/10.1021/ACSOMEGA.5C05523.
